# Preparedness for dental practice in Australia: a qualitative study on the experiences of final-year students and new graduates

**DOI:** 10.1186/s12909-023-04306-0

**Published:** 2023-05-08

**Authors:** Rodrigo Mariño, David Manton, Katharine Reid, Clare Delany

**Affiliations:** 1 Melbourne Dental School, University of Melbourne, Melbourne, Australia; 2 Center for Research in Epidemiology, Economics and Oral Public Health (CIEESPO, Faculty of Dentistry, Universidad de La Frontera, Temuco, Chile; 3 University of Groningen, UMCG, Centre for Dentistry and Oral Hygiene, Groningen, The Netherlands; 4 Department of Medical Education, Melbourne Medical School, University of Melbourne, Melbourne, Australia

**Keywords:** Oral health, Oral health professionals, Preparedness to practice, Student voice

## Abstract

**Background:**

Limited data exists regarding the perspectives of newly graduated dental practitioners (NGDPs) and final-year students (FYS) about their preparedness for dental practice. This information is crucial to inform developments in ongoing professional development for newly qualified dental practitioners and future reviews and development of accreditation standards, policies, and the professional competencies of newly qualified dental practitioners. Thus, the primary goal of this paper was to describe the perceptions of preparedness for dental practice of NGDPs and FYSs.

**Methods:**

Individual semi-structured interviews were conducted between March and July 2020. All interviews were audiotaped, transcribed, and analysed using a thematic analysis.

**Results:**

Eighteen NGDPs and four FYS from across Australia participated in the qualitative interviews. A strong theme from the data was that respondents believed they were well prepared for common challenges in dental practice and patient care. A second prominent theme was participants’ awareness of specific areas of limitation in their knowledge and specific skills including (list them). This data highlights a high level of self-awareness and potential for self-directed learning of NGDPs. It also provides specific content areas for future curriculum developers.

**Conclusions:**

Newly graduated dental practitioner and final-year student participants were satisfied with the theoretical and evidence-based information in their formal learning and teaching activities to begin practicing as dental practitioners. In some areas, NGDPs felt underprepared, mostly attributed to limited clinical treatment exposure, and other contextual elements of clinical practice, and thought transitional support may be required. The research reinforces the value of seeking and learning from students’ and NGDPs’ perspectives.

## Introduction

Graduate dental practitioners (‘dental practitioners’) include dentists, oral health therapists, dental therapists, dental hygienists, and dental prosthetists. Expectations of their professional role are increasing in line with technological and regulatory changes and changing expectations from consumers of health services [1]. All dental practitioners are expected to have attributes of professionalism and skills in critical thinking, communication and health promotion. These are now internationally established components of higher education competencies for health and other professionals [2–5]. However, there is ongoing discussion as to whether dental practitioners believe they have received adequate training in these areas. Dall’Alba, for example, suggests that while skills, knowledge and the ability to conceptualise and problem solve are important, what is fundamental is “students’ capacity to interpret a situation and essentially think and act as a professional practitioner” [6].

There is a paucity of knowledge about whether current dental curricula adequately train dental students for effective collaboration, responsiveness to the changing expectations of health consumers and ultimately, readiness to be in dental practice. Other concerns include whether dental education programs are overly reliant on ‘silo’ teaching which may not prepare students to apply more complex procedures, to be resilient and responsive to others, or to draw from concepts of professional responsibility and ethics [1].

Identifying and evaluating readiness for professional health practice is clearly a complex and multifaceted task. Our previous studies [7, 8] examined, from the perspectives of Australian-based educators, employers, and consumers, whether including these broader professional skills in dental education are preparing newly qualified dental practitioners to meet enhanced expectations.

The primary goal of this qualitative study is to describe Australian newly graduated dental practitioners (NGDPs) and final-year dental professional students’ (FYSs) perceptions of their own preparedness for dental practice. Privileging and separating the voices of FYS and NGDPs from other stakeholders was a deliberate methodological strategy based on research showing the importance of including them as participants, partners, or even agents of change in determining the quality and professional practice relevance of higher education [9–13]. A secondary goal was to combine these data with previous studies of stakeholder views of preparedness to triangulate and strengthen the validity of this information [14].

## Methods

Ethics approval for the research was obtained from The University of Melbourne Human Research Ethics Committee (ethics ID. 1,954,334.3).

Invitations were emailed to FYSs to participate in an in-depth interview via either their dental school or the Australian Dental Students Association; NGDPs were invited to participate via an email from oral health professional associations (i.e. Australian Dental and Oral Health Therapists’ Association, Dental Hygienists Association of Australia, Australian Dental Association, and Australian Dental Prosthetists Association).

Individual semi-structured interviews were conducted by a single member of the research team between March and July 2020. The interview themes were developed with reference to the findings of our previous quantitative study [7], and key themes in preparedness for practice for dental professionals identified in the literature [4]. An interview guide was developed collaboratively by the research team with a research advisory panel specific to these groups (Table 1).

**Table 1:** Qualitative themes covered within the interviews to newly graduated dental practitioner and final-year students

1) Self-assessed levels of preparedness for practice by new graduates
a) Prompt: How would you assess your readiness for practice as a newly graduated oral health professional?
2) Areas particularly felt by you are well prepared for.
3) Areas particularly felt by you are less prepared for.
a) If so, which area(s)? For example, (i) identifying signs of neglect and abuse; and (ii) managing individuals with disabilities or special needs.
4) Clinical areas that new graduates expressed some uncertainty about.
a) Prompts: In managing, for example: (i) medical emergencies (ii) dental emergencies (iii) dental trauma (iv) are there others?
5) Concern about how new graduates felt about practicing as oral healthcare professionals.
a) Prompts: For example: (i) the interface between clinical practice, (ii) patient care and legal obligations (iii) explaining treatment costs, negotiating fees and the financial pressures of business (iv) managing time and stress, balance. (v) others concerns?

All interviews were audiotaped with permission and transcribed with no grammatical corrections for further analysis using Go Transcribe (https://go-transcribe.com/) for further analysis. The authors met online regularly over a period of six months in 2020 to ensure procedural consistency and to discuss themes arising from the data. All data were stored securely and organised via the qualitative data analysis software tool NVivo version 12 (QSR International Pty Ltd, Vic, Australia).

The data were analysed using thematic analysis to identify commonly occurring themes and patterns about NGDPs and FYSs perceptions of readiness for practice [15]. Initial identification of coding categories was undertaken through a systematic reading of the transcribed interviews. Drawing from the seven higher order categories, described by Mahon and Ravindran [4], these categories were coded and organised into key themes and compared within and across themes. Discussion among authors served to affirm coded instances, with discrepancies resolved through group discussion.

## Results

Eighteen NGDPs dental practitioners and four FYSs (two dental and two dental prosthetist students) from across Australia participated in the qualitative interviews. NGDPs included seven dentists, six oral health therapists (OHT); two dental hygienists (DH); and three dental prosthetists (DP). Regarding geographical location, eight were from Queensland; seven from Victoria, two from Tasmania; and one from Western Australia. Nine participants indicated urban locations; eight reported working in regional/rural locations; and one NGDP reported both. Employment history information included practice types—twelve reported private practice only; five reported working in public practice only; and one NGDP reported both (Table 2).

**Table 2:** Characteristics of newly graduated dental practitioner and final-year students interviewed for this study

Qualifications	Year of graduation	Sex	State of study	Practice Descriptor Sector	Practice Descriptor Urban/Rural
Dentist	2019	Male	Victoria	Public	Regional/rural
Dentist	2019	Female	Victoria	Public	Regional/rural
Dentist	2017	Male	Victoria	Private	Regional/rural
Dentist	2017	Female	Victoria/NSW	Private	Regional/metro
Dentist	2019	Male	Tasmania	Private	Regional/rural
Dentist	2018	Male	Queensland	Private	Metro
Dentist	2018	Female	Tasmania	Public	Metro
Oral Health Therapist	2019	Female	Queensland	Private	Regional/rural
Oral Health Therapist	2019	Female	Queensland	Public	Regional/rural
Oral Health Therapist	2019	Female	Victoria	Public	Metro
Dental Therapist	2017	Female	Queensland	Private	Metro
Oral Health Therapist	2018	Female	Victoria	Private	Metro
Oral Health Therapist	2018	Male	Victoria	Private/public	Metro
Dental prosthetist	2018	Male	Queensland	Private	Metro
Dental prosthetist	2018	Male	Queensland	Private	Regional/rural
Dental prosthetist	2018	Male	Queensland	Private	Metro
Oral Hygienist	2019	Female	Queensland	Private	Regional/rural
Oral Hygienist	2018	Female	WA	Private	Metro
Final-year dental students					
Dentist		Female	WA		
Dentist		Male	NSW		
Dental prosthetist		Female	Queensland		
Dental prosthetist		Male	Queensland		

*NSW* New South Wales, *WA* Western Australia

Following an analysis of the interviews, it was considered that thematic saturation was reached, and recruitment ceased. Themes from the interviews were regrouped into six higher-order categories, corresponding to the dimensions of preparedness for practice as identified by Mahon and Ravindran [4].

### Academic and technical competencies

Overall, the theme of feeling well prepared for practice in core dental competencies emerged strongly. Participants reported positive educational experiences and largely believed that they were prepared for initial practice. NGDPs believed that their training prepared them well to join the workforce and provided a sound foundation for safe practice:

*“I think [the university] did actually a really good job in terms of cementing that into [their] grads.” (NGDP6)*

There was also a sense amongst participants that their final-year placements, and the theoretical aspects of the course, prepared them well and provided confidence in commencing practice. Several graduates identified the transition from supervised practice to wholly independent practice as daunting; particularly at the beginning of their practice, due to a strong reliance on demonstrators and supervisors during training, or a perceived lack of sufficient exposure to specific clinical procedures.

*“… just being independent and all that, that's always a big jump, but I think, again, the university has prepared me pretty well for it.” (NGDP11)*

NGs recognised that they had theoretical knowledge but were uncertain whether such preparation would be adequate. They demonstrated high levels of self-awareness by acknowledging that not everything could be taught in the program and resources could be accessed for self-learning.

*“There always will be things that you have not been exposed to and things like that. Also, that dental school has sort of provided us sort of resources and places to go to when we do find those situations.” (NGDP1)*

NGs identified some dental programs as being proactive in providing learning opportunities:

*“… if you wanted something [dental procedures], you would generally be able to swap [dental procedures] and we had a system where students were able to put down on the list things that they hadn’t had an opportunity to do.“ (NG16)*

Structural issues were mentioned as barriers to developing clinical competencies. These barriers went beyond exposure to information, resources, or the quality of demonstrators and academics:

*“…There is a lot of students, big classes, not enough chairs and having that time cut.” (NGDP5)*

A common theme from NGDPs, was a desire for more exposure to practical issues, more laboratory time, hands-on skills, including clinical restorative aspects such as endodontics, prosthodontics (both fixed and removable), as well as paediatric dentistry, and some surgical interventions (dental extractions and implants).

NGs also desired more exposure to aspects of patient care beyond specific oral health complaints. Some believed that more learning opportunities were required on how different aspects of a patients’ life influenced oral health care to encompass a more holistic view of the overall health of patients:

*“…I think just in the curriculum in general, not focusing just on the physical, but also a greater emphasis on how socioeconomic things or disadvantaged culture, family types, that sort of thing, influences treatment.” (NGDP5)*

NGs also suggested more specific teaching around recognising the signs of neglect and domestic abuse, as concern existed that not all areas where abuse or neglect may occur were covered (e.g., children or older adults). NGDPs and FYSs identified the need to have clearer protocols and guidelines on how to deal with these issues. Another area of concern for participants was the relatively low exposure to managing special needs patients.

### Communication and interprofessional skills

Participants regarded communication and interprofessional skills as important competencies and overall felt that their communication skills of graduate dental professionals were acceptable for practice. Although they were aware that theory helped to improve their communication skills, they also highlighted that a practical approach to communication skills training would likely be more beneficial:

*“A lot of people just do it in a very robotic way, not in a way that you will. I don’t think I’ll carry it into practice. They’re only doing it now because it’s on the form and they’re told they have to read it” (FYS3)*

NGDPs were especially tested by communicating with patients perceived to be ‘challenging’ to communicate with (e.g., difficult, or abusive patients). Some NGDPs believed that they were unprepared to communicate with a parent or guardian, due to age differences, or where there were cultural and linguistic diversity, special needs and mental health concerns.

Moreover, NGDPs suggested extending communication skills training to cost-of-care and treatment plan discussions with the patient to obtain the best possible clinical outcome and referral communications. This same observation was made in relation to clinical entrepreneurship and financial solvency.

These observations show that NGDP’s were aware of both their own limits to knowledge and skills and also aware of the challenges to incorporate broader communication and skills training into the clinical training at dental schools.

### Protective mechanisms and adaptative skills

Newly graduated dental practitioners articulated a range of coping strategies used in work situations, including managing time, everyday stressors, and balancing work and personal life. One NGDP identified the potential for increased student support during the course, particularly with respect to mental health and well-being and maladaptive strategies in their early years in practice:

*“I think mental health-wise, it probably needs a bit of improvement, to be honest, because I find that a lot of students who I knew, who had a lot of potential, just resort to either dropping out of the course or choosing something completely different, or feeling like they have to harm themselves or they're unable to talk to people…” (NGDP5)*

Examples of maladaptive strategies that some NGDPs had adopted included self-blame, self-doubt and self-criticism:

*“I used to always focus on what went wrong, but I never gave myself credit for what went right or what worked. And I was always making lists of things that I could change rather than things that worked…” (NGDP5)*

For others it was more a matter of gaining confidence while applying what they had learned during their training.

*“… I think a lot of it is just self-doubt. Self-doubt that probably is not warranted in the beginning because you have been trained in a good program and you have good experience. Enough to get into workplace and practice safely.” (NGDP6)*

### Professional attitude and ethical judgement

Participants identified self-reflection on their own practice and professional competence as the most significant issues in their preparedness for practice. Professional competence was considered both from the “*what am I able to do based on my training*” and “*I’d like to try it to learn it*” perspectives:

*“And I feel like we were definitely told, “this is your scope of practice”. Like you only do what's in your scope, what you’ve being trained in and are competent in”. (NGDP4)*

FYSs’ self-reflections as future practitioners also recognised their limitations, while simultaneously, trying to extend themselves within their scope of practice:

*“I think it's going to be a pressure of trying to achieve a balance between being not adventurous, … and we keep growing and developing new skills, but also being somewhat sensible and practicing within the scope about limitations and putting our patients at risk.” (FYS2)*

New graduates were also aware of their limitations as NGDPs and of the need to continuously upskill their knowledge base. They described the need for continuing professional development to keep up-to-date and improve their skills and knowledge.

NGDPs displayed awareness of the legal and ethical frameworks related to their dental professions and understood the value of this domain in delivering effective and safe oral health services. In the context of high levels of awareness, they also described being unprepared to deal with some legal aspects of dental practice:

*“Basically, the teaching of law has been like all of you are going to get a complaint at some point, or all of you are going to get sued at some point. So just accept it and don’t worry about that, kind of thing, which hasn't really put any of us at ease” (FYS3)*

Nonetheless, NGDPs showed that they were able to self-evaluate their own abilities and training to provide safe dental care within their scope of practice.

*“I think I'll always give it a try and give it a shot, but sometimes I do take a step back and have a look and say,’look, that's way beyond my capabilities’” (NGDP11)*

NGs also described a lack of knowledge of their scope of practice by other members of the dental team. Significantly, new OHT, DH, and DP graduates reported that their scope of practice was not well known by the other dental professions, in particular the awareness of OHTs’ and DHs’ by dentists:

*” I know what part of my scope was, but what the dentist thought of my scope was a completely different story at the beginning.” (NGDP14)*

Oral health therapists and DP professionals often reported feeling less valued as a member of the dental care team:

*“… some of the private practice will not treat us like, we are not treated like an equal member in the dental team.” (NGDP15)*

Similarly, new OHT graduates reported that their scope of practice was misinterpreted by other dental professionals and highlighted the need for more dental team emphasis during training:

*“I felt like the university, […] the dental school hasn't prepared well enough to know like our place in the dental team” (NGDP15)*

### Clinical entrepreneurship and financial solvency

Participants identified some challenges in adapting to a business environment in private practice and managing financial responsibility. Both NGDPs and FYSs recognised the role that their training in the public healthcare system played in their impressions of managing the financial aspects of practice. During dental training, this was seen as an advantage, as well as a limitation:

*“…we are at an advantage in the sense that we do not really need to navigate the tricky financial side of things, that we don't need to really adapt our treatment plans accordingly." (FYS2)*

However, NGDPs acknowledged that the contrast between public and private contexts became more evident when NGDPs entered practice. New graduates perceived two different, contrasting ways, to be prepared for practice in oral healthcare. For NGDPs, the discussion of cost in private practice was somewhat daunting. Some graduates deliberately chose to become public dental practitioners to reduce the need for financial discussions with patients:

*“…that's like, honestly the main reason I went into public, because it stressed me out thinking about having to try and justify how much money I'm making at work and just all those types of things.” (NGDP4)*

Both FYSs and NGDPs identified varying levels of training about the business aspects of practice, with an emphasis on their limited exposure to private dental practice in their training. For some NGDPs, financial training was non-existent, or did not meet expectations, and varied according to the NGDPs university and dental profession:

*“…but if it's for a private practice perspective, I don't think any, well, from mine, from [dental school] perspective, I don't think any graduates from [dental school], have received enough training and preparation to get into the private world.” (NGDP15)*

Some NGDPs had some relevant experiences on placements and exposure to a mix of private and public patients during their final years of training. For example, a DP believed that the exposure and training was sufficient preparation for the financial and entrepreneurial aspect of practice:

*“I think there was a dental practice management unit. It was a whole unit for the semester, which explained pretty much everything that could have been required to set up and run a practice there.” (NGDP10)*

Graduates from universities where the training program had an established private clinic, described the value of this exposure for understanding the business elements of dental practice. However, the majority learn these business skills once they graduate and were exposed to daily practice.

### Social and community orientation

The interviews investigated participants’ preparedness related to knowledge and skills in treating patients from a variety of culturally and linguistically diverse backgrounds, and Aboriginal and Torres Strait Islander People. Most participants believed that they received exposure to patients from culturally diverse backgrounds during their training; however, they believed that this exposure was *ad-hoc*:

*“I guess we've got quite a diverse patient base, so I guess we got some passive exposure depending on your patients, of course.” (FYS2)*

Participants believed that cultural training was not sufficiently comprehensive. In fact, some participants considered that most of their cultural and social perceptions evolved from their upbringing, high school and primary school education, and past personal experiences. Many participants believed that the current framing of the cultural competence curriculum was: *“really a box ticking exercise for you.” (FYS3)*

Issues around the provision of care according to the patient’s culture were also discussed. Both NGDPs and FYSs commented that, although they received training to treat people, there was less coverage or emphasis on cultural and psychological aspects of care during training. As a result, NGDPs perceived that they were unprepared to manage patients from culturally and linguistically diverse backgrounds and to provide culturally safe dental services:

*“…things about safety and more about the psychology, understanding emotions and even cultural differences as well, that would be a good addition to the degree, not just looking at the medical aspect.“ (NGDP5)*

Several NGDPs mentioned limited preparedness for practice in relation to social factors and health inequalities:

*I think just in the curriculum in general, not focusing just on the physical, but also a greater emphasis on how socioeconomic things or disadvantaged culture, family types, that sort of thing, influences treatment…” (NGDP5)*

Empathy was also stressed as a major issue by some NGDPs. One NGDP commented that a more wholistic approach to patient care should be addressed or emphasised during dental training:

“They say, my patient is such a big ****, and I’d say well, your patient has gone through a divorce; your patient is under financial strain; your patient has to pay for a taxi to get to the hospital, or she has to walk upstairs and she's on crutches or using a walking stick.” (NGDP5)

## Discussion

Overall, NGDPs and FYSs reported being generally prepared to join the workforce, with their clinical learning making them ready for the more common challenges in dental practice and patient care. On the whole, participants agreed that they received adequate theoretical and evidence-based information in their formal learning and teaching activities. This view accords with the goals of the ADC framework for Australian dental practitioners [16]. This research also highlights that NGDP and student dental practitioners had high levels of self-awareness of their own competency strengths and deficiencies in particular areas of knowledge and skills. Participants’ awareness of the need to keep learning accords with literature that has previously acknowledged that consolidating competencies in clinical practice is a lifelong learning process which can only be achieved with further practice [17].

Despite the overall positive perceptions of preparedness, specific areas were identified in which NGDPs felt they would benefit from further training and consolidation, as well as areas where more experience might be desirable. Consistent with our previous publications [7, 8], NGDPs also identified lower perceived preparedness in the practice areas of managing dental trauma, medical emergencies, identifying and acting on abuse or neglect, and effectively managing patients with special needs. Exposure to such experiences varies as some schools have integrated opportunities to treat patients with special needs, patients in nursing homes and mental health patients [18].

The present findings were also consistent with previous studies of NGDPs [19–21] and FYSs [21], which also reported the least confidence in performing dental procedures similar to those nominated by our participants. Confidence appeared to be a function of both procedure complexity and the amount of clinical experience participants had with that procedure [21]. It was reassuring that despite concerns about the impacts of reduced clinical and laboratory time, NGDPs were aware of their limitations and seemed to have developed self-discipline and ethical awareness that would allow them to acknowledge their own limitations and those conditions/situations when they would not have the experience or expertise to provide safe care.

Furthermore, NGDPs described that evidence-based practice informed their patient care strategies, both in their clinical learning and upon entering practice. Their self-perceived level of professional competence was seen as a way for the NGDPs to ‘protect’ themselves. Several NGDPs (i.e., DH, OHT) believed that their training reinforced that they should not extend beyond their scope of practice (e.g., dental hygienist, oral health therapists) and level of clinical skills, and should reflect on their practice and utilise these perceptions to seek appropriate professional development opportunities to improve their professional skills and competencies.

NGDPs believed that overall, they were well prepared in communication skills with a good theoretical communication background. However, some described less confidence in more challenging aspects of communication where more complex communication skills were required, such as discussing sensitive issues, or discussing financial aspects and situations where more assertive communication would be needed [20–22]. NGDPs believed that their communication for referral pathways needed to be developed further and found discussions with parents could be challenging. These areas were also mentioned by supervisors and employers as an area in which NGDPS were underprepared [8].

Newly graduated dental practitioners also highlighted a lack of knowledge of the scope of practice of other members of the dental team. Usually, an OHT or a DP participant provided an example of where a dentist was unfamiliar with their scope of practice. These NGDPs often felt less valued as a dental team member, which highlights a need for stronger interprofessional education within dental training programs [23]. Given many providers offer multiple dental training programs, there is an opportunity to engage in formalised interprofessional activities to strengthen understanding of the scope of practice of others in the oral healthcare team [23].

New graduates observed that although they were generally comfortable treating patients from culturally diverse backgrounds, they may not be prepared to provide the best practice for the patient’s cultural needs and background. The training received for this aspect of patient care varied from *ad-hoc* and passive learning to more formalised learning. This experience is supported by reviews on the teaching techniques for cultural competence among dental students [24, 25] and from among other dental professional stakeholders’ [8]. Nonetheless, employers of NGDPs perceived that they are far better prepared to address cultural aspects of care than they were themselves [8].

The research findings have implications for training providers. The research highlights the need for educators to focus resources on encouraging their learners to adopt adaptative coping strategies to manage some of the challenges of practice and to address maladaptive behaviours such as self-blame and self-criticism [22]. Maladaptive behaviours among dental trainees may be associated with the high-achieving nature of dental programs and/or perfectionist mindsets that may be ill-suited to dental practice. Resources could also extend to support for learners and NGDPs to manage any mental health issues that may become more problematic during clinical learning or upon entering the workforce.

Interestingly, although our study was undertaken during the COVID-19 pandemic, the use of Information and Communication Technology (ICT) (e.g., telehealth) was only rarely mentioned by NGDPs as a tool for communication and exchange of information with colleagues and patients. However, these and other developments already in operation in healthcare, are transforming the delivery of healthcare and will require the development of new competencies amongst dental practitioners [26–29].

The limitations of this study include the self-reported nature of the responses, that may have either overstated or understated assessments of preparedness for practice. Additionally, the invitation to participate was sent just prior to the COVID-19 pandemic lockdown (late February 2020) in many parts of Australia, which may have affected peoples’ willingness to participate. Despite these limitations, the study achieved broad representation in terms of dental professions and dental schools, suggesting that the present findings could contribute significantly to current knowledge and could be transferable to other jurisdictions with some degree of confidence. Another limitation was the small number of FYSs. FYSs reflected on their experiences as future practitioners and their responses were similar overall to NGDPs; however, FYSs and NGDPs have quite different perspectives; with one group having not experienced independent practice. Nevertheless, this study privileged the voice of students and NGDPs and used semi-structured interviews which provided a depth of participant experience to complement what is known from quantitative studies [8, 12]. Still, future studies should further explore FYSs’ perceptions of preparedness to practice.

A key goal of this study was to show how NGDPs perceptions of preparedness to practice aligned with or were contrary to other stakeholders’ assessment of competence, as a lack of convergence might lead to inappropriate expectations of performance [30]. Interestingly, the responses of NGDPs were generally convergent with those of dental educators and clinical supervisors across quantitative and qualitative methodologies [7, 8]. Any differences were more in the strength of the response, rather than the direction, which generally reflected that NGDPs met appropriate performance standards.

## Conclusion

The primary goal of this paper was to describe the perceptions of preparedness for dental practice of NGDPs and FYSs. The study, supported by other study findings [7, 8, 13, 20, 31, 32], shows highly consistent findings that dental practitioners are generally well prepared for practice. This research also identifies and distinguishes between areas in dental training that could be improved and areas achieving appropriate standards. This study demonstrates a high level of self-awareness in students and new graduates of limits to their knowledge and skills, and thus reinforces the value that seeking feedback and perspectives from students as to their learning needs. It is hoped that these findings will provide dental educators and curriculum developers with an overview of how NGDPs and FYSs assess their preparedness to practice in the Australian context and the evidence needed to inform developments in educational programs for dental practitioners and professional development for newly qualified dental practitioners.

## Data Availability

The datasets generated and/or analysed during the current study are not publicly available due to the ethics approval granted on the basis that only researchers involved in the study can access the de-identified data. The minimum retention period is five years from publication. Supporting documents are available upon request to the corresponding author.

## Abbreviations

ADC: Australian dental council
NGDP: Newly graduated dental practitioner
FYS: Final-year students
OHT: Oral health therapists
DH: Dental hygienists
DP: Dental prosthetists
